# The landslide’s conceptualizing economic decline and its framing effect: Mandarin evidence

**DOI:** 10.3389/fpsyg.2023.1271911

**Published:** 2023-10-30

**Authors:** Yan Zhang, Wenxing Yang

**Affiliations:** College of International Studies, Yangzhou University, Yangzhou, Jiangsu, China

**Keywords:** corpus, metaphorical framing, economic decline, natural disasters, landslide frame

## Abstract

Conceptual metaphors are essential for explaining and understanding social concerns. Natural disaster metaphors are commonly employed to access the abstract and negative impacts of social issues. Five of the top 10 most prevalent natural disaster frames in the Center for Chinese Linguistics (CCL)—earthquake, flood, fire hazard, drought, typhoon, landslide, volcano, sandstorm, tsunami, and debris flow—share a common economic target domain and show economic recession. Additionally, corpus-based research has revealed that the landslide frame is the most salient in figuratively representing economic declines. An experimental study derived from the corpus analysis has found that the landslide-framed economic crises posed more severity to participants and exerted a notable influence on their opinions and judgments. Therefore, when effective communication of economic hazards is to be realized, metaphorical representation of economic crises demands great consideration.

## 1. Introduction

Recent years has seen a focus on risk warning and communication as the COVID-19 epidemic and periodic extreme weather have shown the effects of these events on humanity. The study and communication of risk have received increasing attention from academics and organizations (e.g., World Health Organization, 2020; Hyland-Wood et al., 2021; Tang, 2022). Scholars concur that in the case of rising dangers, effective, concise, clear, and official communication of risk sources, symptoms, and confident solutions is urgently needed (Hyland-Wood et al., 2021). Generally, communication about risks or crises is carried out through language, whether it is verbal or non-verbal.

More than just a literary device, metaphor is a method of thinking that permeates all aspects of our lives and structures our thoughts (Lakoff and Johnson, 1980). Metaphors are occasionally used in risk communication to grasp abstract ideas in terms of more concrete ones. According to conceptual metaphor theory and embodied philosophy (Johnson, 1987), understanding and reasoning are realized through mappings from concrete concepts (source domains) to abstract ones (target domains), particularly from perceptible concepts and sensorimotor experiences derived from daily life such as spatial orientation, containment, force, and temperature to imperceptible ones such as time, emotions, and power. According to prior research, metaphorical frames such as *organisms* and *natural disasters* are typically used to communicate economic crises or declines (e.g., Charteris-Black and Ennis, 2001; Charteris-Black and Musolff, 2003; de los Ríos, 2010; Wang et al., 2013; Piromalli, 2021; Zeng et al., 2021). Recent psychological research has implied the exertion of metaphorical framing effects on reasoning, i.e., the persuasive power of metaphors (e.g., Thibodeau and Boroditsky, 2011, 2013, 2015; Thibodeau, 2017; Thibodeau et al., 2017; Thibodeau and Flusberg, 2017; Joris et al., 2019; Hart, 2021; Benczes and Ságvári, 2022; Brugman et al., 2022; Tao et al., 2023). Some psychological researchers argue that metaphors shape reasoning in the way that metaphor frames appear to instantiate frame-consistent knowledge structures and invite structurally consistent inferences, thus affecting reasoning and opinions (e.g., Charteris-Black, 2011; Thibodeau and Boroditsky, 2011, 2013, 2015; Landau et al., 2014; Thibodeau et al., 2017). In this study, we seek to identify the metaphorical framing effect of the most salient natural disaster—landslide frame (elicited from corpus analysis) in Mandarin—when conceptualizing economic decline or crises. A metaphorical framing study into economic decline may reveal the persuasive power of metaphors, thus providing earlier warning of risks and better communication for the wellbeing of the general public.

### 1.1. Metaphors of economic crises

Clusters of metaphors render economic concepts graspable, accessible, and vivid. Many scholars show great interest in such linguistic phenomena. Charteris-Black (2000) summarizes metaphors of the economy through a corpus analysis of *The Economists* and reveals that *organization, people, and animal* are the most frequent metaphors. More economic crises metaphor analysis is revealed by scholars such as Charteris-Black and Ennis (2001) and Charteris-Black and Musolff (2003). They made comparative economic crises metaphor studies in English and Spanish, as well as English and German financial reporting, respectively. Based on their investigation, English and Spanish share metaphorical mappings from *organism, physical movements, and natural disasters* to the economy and market movements. English and German share mappings from *up-and-down movements* in trading to health frames. Furthermore, de los Ríos (2010) wraps up metaphors for the economic crises with images from *The Economist*’s seven covers, including *weather, natural disasters, and end-of-the-world* scenarios. The metaphors in play are as follows: *The economic crises is an earthquake shaking Wall Street; the savings bank economic woes are twin tornadoes; the economic crises is a whirlpool, a credit drought*, and *climatic conditions*. Piromalli (2021) conducted a thorough analysis of the two main metaphors for the economic crises: *metaphors for illness and metaphors for natural disasters*. Under the same conceptual mapping, ECONOMY IS ORGANISM, Wang et al. (2013) compared metaphors used to describe the economic crises in 2008 in British and Russian economic discourse: an *economic crises is a living or sick organism*. As seen from the above studies, the following frames for economic crises stand out: *organism, object, and natural phenomenon*.

### 1.2. Metaphorical framing

The frame needs clear clarification when it comes to the metaphorical framing effect. According to Ritchie (2013), a frame is a pattern of language use and a cognitive schema. The definition of framing, which is frequently cited, is as follows:

Framing essentially involves *selection* and *salience*. To frame is to s*elect some aspects of a perceived reality and make them more salient in a communicating text, in such a way as to promote a particular problem definition, causal interpretation, moral evaluation, and/or treatment recommendation* for the item described. (Entman, 1993, p. 52)

Frame emphasizes frame-congruent features in communication (Entman, 1993). For instance, the COVID-19 pandemic’s antagonistic, evil, hazardous, and urgent nature is highlighted when the pandemic is depicted as a war. A dramatic war scenario would inspire a sense of urgency and community and successfully persuade people to abide by rules and regulations (Charteris-Black, 2021). Entman (1993) does point out that metaphorical frames would leave out metaphorical targets’ frame-incongruent characteristics. Thus, when COVID-19 is metaphorically framed as a battle, the necessity of finding the source, the recovery phase, and maintaining social distance are all left out (Semino, 2021). According to Semino et al. (2018), framing is a function of metaphor and has roots in psychology and sociology (Pan and Kosicki, 1993). These perspectives are cognitive, discursive, and practice-based (Semino et al., 2018). Meanwhile, metaphorical framing echoes the views of de Severino et al. (2001) concerning the functions of metaphor: (1) to manipulate readers’ minds through the inference patterns and value judgments generated by metaphors and (2) to give a more concrete representation of the situation at hand, making it clearer.

Metaphors are found to be 6% more influential and persuasive than literal language in persuasion tasks when metaphorical framing is observed in practice (Sopory and Dillard, 2002). Recent psychological studies suggest that metaphorical frames affect people’s opinions and judgments by eliciting knowledge structures that are consistent with the frame and inviting structurally consistent inferences (e.g., Robins and Mayer, 2000; Schlesinger and Lau, 2000; Charteris-Black, 2011; Thibodeau and Boroditsky, 2011, 2013, 2015; Landau et al., 2014; Thibodeau et al., 2017; Thibodeau and Flusberg, 2017; Joris et al., 2019; Hart, 2021; Benczes and Ságvári, 2022; Brugman et al., 2022; Tao et al., 2023). People may propose different solutions to the same social issue—crime—in studies with a framing effect of significant difference (Thibodeau and Boroditsky, 2011, 2013). Participants have a strong tendency to think that it is best to strengthen law enforcement and punishments for criminals when a crime is referred to as a beast. When crime is portrayed as a virus, reforming social administration is frequently suggested. When cancer is metaphorically framed as a journey and a battle, patients adopt different emotions and mindsets. When cancer is incurable, the battle metaphor would make patients feel more guilty than the journey metaphor (e.g., Hendricks et al., 2018). However, a follow-up study by Steen et al. (2014) was conducted due to inconsistent results with the earlier study. Similar-sized but modest metaphorical framing effects would result from metaphors in various communication modalities (Flusberg et al., 2020). When examining the impact of COVID-19’s war and sports metaphors on feelings and thoughts in the era of pandemics, de Saint Preux and Blanco (2021) note a weak metaphorical framing effect. Even so, the difference is not statistically significant.

In an economic discourse, Joris et al. (2019) discuss participants’ attitudes toward the Euro crises when it is metaphorically framed as war and disease. The results demonstrate that participants take on a metaphorical frame-congruent evaluation of the Euro crises. Participants in war conditions significantly more often refer to war when answering the open questions. On the contrary, when the Euro crises is framed as a disease, participants tend to use words and sentences containing disease-frame elements. In the case of anti-telefraud communication, when telefraud is framed as disease, war, or issue, the war-framed telefraud poses more severity than the issue-framed one to participants without fraud experiences. However, this study also showcases the metaphorical framing effect as well as potential factors influencing the framing effect (Liu and Chen, 2023).

A comprehensive study of the aforementioned metaphors of different frames for crime (Thibodeau and Boroditsky, 2011), depression (Reali et al., 2016), cancer (Hendricks et al., 2018), COVID-19 (de Saint Preux and Blanco, 2021; Zhang et al., 2022), migration (Benczes and Ságvári, 2022), anti-immigration (Hart, 2021), climate change (Flusberg et al., 2017), telecom fraud risk (Liu and Chen, 2023), price trend (Morris et al., 2007), and their respective strong, weak, or non-existent framing effects demonstrates that the results of metaphorical framing effect can vary and are inconsistent, which necessitates further research into this linguistic and psychological phenomenon.

### 1.3. Current research

Departing from the gist of embodied cognition, natural phenomena are the most familiar ones for their omnipresence in weather conditions, our dependence on nature, and our interaction with nature over the years since our human being’s existence. However, previous studies limited their research to economic metaphors in English, German, Russian, etc. There is a shortage of systematic analysis of economic decline or crises metaphors framed by natural disasters in Mandarin. Therefore, our first research question goes as follows: Are natural disaster frames equally and frequently employed in comprehending economic conceptions in Mandarin? If so, what is the most frequent natural disaster frame in the economic crises metaphor? To achieve the end, we focused on the economic crises metaphors of natural disaster frames in Mandarin by carrying out a corpus-based analysis in the next section.

Given that the high frequency of economic crises metaphors framed in natural disasters prevails in Mandarin contexts based on the corpus analysis, we were questioning whether economic crises metaphors of different frames would have an impact on people’s psychology and judgments and even alter their behavior, which is of great importance in risk warning and communication in the economy field and the general public’s economic behavior. Therefore, our second research question is whether differently framed economic crises metaphors influence participants’ conceptualization and judgments of economic crises. To find the answer, an experiment was designed to investigate whether economic crises in a literal and one specific natural disaster frame would give rise to different conceptualizations and lead to different opinions, behaviors, and judgments. The natural disaster frame involved in this part was the one with the highest metaphorical frequency emerging from the corpus analysis in the first research question. Based on the metaphorical framing effect hypothesis, we hypothesized that literal and natural disaster-framed economic crises would shape participants’ reasoning about economic crises differently and lead to different economic behaviors and judgments.

## 2. A corpus analysis: study of natural disaster-framed economic decline in CCL

According to the theories of embodied cognition (Lakoff and Johnson, 1980; Johnson, 1987; Lakoff, 1987), metaphors that are framed in the context of natural phenomena are prevalent. Examples include brainstorming, a shiny person, a depressing mood, and a financial tsunami. According to research on the conceptualization of economic crises in English, Russian, German, and Spanish (e.g., Charteris-Black and Ennis, 2001; Charteris-Black and Musolff, 2003; Morris et al., 2007; de los Ríos, 2010; López and Llopis, 2010; Wang et al., 2013; Piromalli, 2021), natural disaster frames are frequently used. In this section, we adopted corpus-based analysis to identify which specific natural disaster frame was most frequently used to discuss ideas of economic decline in Mandarin Chinese.

### 2.1. Methodology

#### 2.1.1. Data sources

We selected CCL as the research corpus to identify the top 10 natural disaster frames. CCL, an unprocessed corpus with 783,463,175 tokens made up of raw material from networks, books, and news reports, offers a comprehensive database from the 11th BC to the present for the study of Chinese. This corpus acts as a precise and thorough database for the study of Chinese language phenomena. The 22 most frequent natural disaster frames in China were entered into CCL as follows: storm (暴风雨), earthquake (地震), tsunami (海啸), cold wave (寒潮), flood (洪水), volcano eruption (火山), hurricane (飓风), tornado (龙卷风), typhoon (台风), debris flow (泥石流), landslide (滑坡), drought (干旱), hail (冰雹), sand storm (沙尘暴), snowstorm (暴雪), fire hazard (火灾), red tide (赤潮), frost damage (霜冻), sleet (冻雨), haze (雾霾), thunderstorm (雷暴), and heat wave (热浪) (Wang, 2006). After ranking all the frequencies of 22 natural disaster frames, the following 10 frames were the most frequently used in Mandarin and would go through our detailed corpus-based analysis: *earthquake, flood, fire hazard, drought, typhoon, landslide, volcano, sandstorm, tsunami, and debris flow*.

#### 2.1.2. Data analysis procedures

Prior to the corpus analysis, we consecutively went through the following procedures concerning 10 natural disaster frames: (1) entering and searching a natural disaster frame into CCL; (2) downloading all the hits to a text file; (3) uploading the text file into the corpus analysis tool AntConc; (4) extracting the collocation and KWIC of the natural disaster frame; (5) excluding all the literal usage of the frame; (6) identifying the metaphorical target domain based on the Metaphor Identification Procedure (MIP) developed by the Pragglejaz Group (2007) and referring to context and the *Comprehensive Dictionary of Chinese Language* (汉语大词典). Despite the linguistic data of CCL starts in the 11th BC, all the sentences, including these 10 natural disaster frames, were downloaded, whether they originated from ancient times or contemporary days, going through the above procedures.

### 2.2. Findings and discussion

Metaphors serve as a great bridge between perceptible phenomena and imperceptible notions, say natural disasters and economic decline. As shown in Table 1, apart from their literal use, natural disaster frames take on metaphorical uses in various fields such as health, management, economy, politics, society, education, relationships, mindsets, and emotions, which literally cover every aspect of humanity’s life. After careful mapping identification between natural disasters and various target domains, the metaphorical use of natural disaster frames in the economy domain stands out for its prominence and high frequency of occurrence in 7 of the 10 frames of Chinese contained in Table 1. A more specific target domain of economic decline can be metaphorically understood in five natural disaster frames (earthquake, flood, landslide, tsunami, and debris flow), which again illustrates the gist of embodied philosophy that abstract concept understanding is deriving from daily embodied experiences (Lakoff and Johnson, 1980).

**Table 1:** Overview of 10 natural disaster frames by overall, metaphorical frequency, and percentage of metaphorical use; provided with illustrative examples in CCL.

Frames	Total hits	Metaphorical freq.	% metaphorical	Target domain frequency and examples
Earthquake (地震)	12,515	106	0.85%	Turmoil in politics (39), sports (7), **economic and financial (16)**, human resources (22), relationship (22)
Flood (洪水)	10,615	269	2.5%	crowds and emotion eruption (116), **shock in economy (17)**, politics, disease and science development (136)
Fire hazard (火灾)	7,498	1	0.01%	Problems (e.g., end the awkward situation of relying on America to put out the fire hazard.)
Drought (干旱)	5,222	1	0.02%	Industrial development (e.g., shortage in capital and drought in industries)
Typhoon (台风)	4,008	21	0.5%	Turmoil in politics and society (10), **new trends in economy** (1) and thoughts (6), rapid execution of the plan (4)
Landslide (滑坡)	3,763	2013	53.5%	**Decline in: economy (1372)**, health and environment (44), quality and management (125), morality and values (188), arts and education (284)
Volcano (火山)	2,829	198	7%	Emotions, desires, and passions eruption (123); volume of voice and crowds (34), **economic risks** and **chances (41)**
Sandstorm (沙尘暴)	1877	8	0.43%	Erosion of morality (3), erosion of cultural values (3), blame and abuse (2)
Tsunami (海啸)	1,592	128	8%	**Financial crises (60)**, mental struggle (21), big volume of voice (43), political turmoil (4)
Debris flow (泥石流)	1,473	6	0.41%	**Downward movements of economy (3)**, loss of intellectual talents (3)

The bold terms in table highlight the shared target domain for these ten natural disaster frames.

Aside from their similar metaphorical mappings in the economic domain, it is important to note that each of these five natural disaster frames represents a different degree of negative economic impact, with the landslide and tsunami frames, according to context analysis, being the ones closest to an economic crises. In spite of the shared economy domain between the seven frames, the TYPHOON frame shows a chance for economic growth, and the VOLCANO frame represents money entering and exiting the stock market, showcasing its neutral characteristics. In order to demonstrate a metaphorical economic decline in natural disaster frames and identify the most prominent metaphorical frame of economic crises, these two frames (TYPHOON and VOLCANO) would be excluded from our analysis of the frames that came after them.

#### 2.2.1. Earthquake frame

以石油为主要经济来源的海湾国家遭受了一场严重经济“[**地震**]”, 经济形势严峻。【文件名:\当代\报刊\人民日报\1998年人民日报】.

*Translation: Gulf States, whose petroleum is the main source of their income, suffered from an economic **earthquake** and were confronted with severe economic situations (File Name:\Contemporary\Newspapers\China Daily\China Daily, 1998).*

地震 (dì zhèn) “earthquake,” a prominent natural disaster with its biggest frequency in the CCL corpus, attacks the whole world with shaking of the ground caused by seismic waves, threatens the earth inhabitants with its suddenness and unpredictability, and causes great loss and casualties every year all over the world. In this example, the economic turbulence in the Gulf States is framed as an earthquake in the metaphor “ECONOMIC TURBULENCE IS EARTHQUAKE.” The suddenness and unpredictability, as well as the losses, and casualties caused by the earthquake, are perfectly mapped into the economic losses and turbulence in the Gulf States.

#### 2.2.2. Flood frame

现有的垄断就会受到新进入者或该行业中原有边际厂商扩张引起的[洪水]般的冲击。【文件名:\当代\CWAC\CPB0223.txt】.

*Translation: The existing monopoly is then subject to a [flood] of shocks caused by new entrants or by the expansion of the original marginal players in the industry. (File name:\ Contemporary\ CWAC\ CPB0223.txt).*

洪水 (hónɡ shuǐ) “flood,” featured by enormous overflowing of water soaking or drowning the land, usually bringing destructive influence on crops and properties. In the quoted example, the massive scale of shock and damaging features of the flood are mapped onto the great negative economic shock received by the existing monopoly in the metaphor “ECONOMIC SHOCK IS FLOOD.”

#### 2.2.3. Landslide frame

工业生产总值已大大超过了农业生产总值, 农业开始呈现出[**滑坡**]的趋势。【文件名:\当代\史传\晚年蒋经国】.

*Translation: Total industrial output exceeds agricultural ones greatly, and agriculture is taking on a tendency toward **landslides**. (File Name:\Contemporary\Historical Biography\Jiang Ching-kuo in his later years).*

滑坡 (huá pō) “landslide” refers to a downward movement of earth, rocks, debris, and so on, which usually brings danger to lives right below the landslide and causes blocking and traffic congestion. The landslide frame in this example conveys downward and dropping features perfectly for the production of agriculture. Therefore, “BUSINESS RECESSION AND DECLINE IS LANDSLIDE” serves as a metaphor for natural disaster landslides and agriculture production.

#### 2.2.4. Tsunami frame

在这场危及世界金融安全的全球性金融[**海啸**]面前, 每个国家都会把捍卫本国的金融利益放在首位。【文件名:\当代\网络语料\网上面试笔试题】.

*Translation: Faced with a financial **tsunami** threatening global financial security, safeguarding financial interests would be the top concern of every country (File Name:\Contemporary\ Network Corpus\ Questions for Network Interview).*

海啸 (hǎi xiào) “tsunami,” a catastrophic ocean wave usually caused by a submarine earthquake, an underwater or coastal landslide, or a volcanic eruption, brings great danger and even drowns and ruins to the coastal areas with enormous waves. In the above sentence, the aftermath of the tsunami is conferred on global finance so as to give rise to a financial crises in the economic field, with the metaphor “ECONOMIC CRISES IS TSUNAMI.”

#### 2.2.5. Debris flow frame

这是一场脱离基本经济因素倾向的市场投机力量所造成的“[**泥石流**]冲击”【文件名:\当代\报刊\1995年人民日报】.

*Translation: This is a **debris flow/mudslide** shock derived from the force of market speculation deviating from economic fundamentals (File Name:\Contemporary\Newspapers\ China Daily\China Daily, 1995\June).*

泥石流 (ní shí liú) “debris flow” is defined as slurry flows consisting of sediment-water mixtures incorporating fine material (sand, silt, and clay), coarse material (gravel and boulders), and a variable quantity of water (Elias and Alderton, 2020). Debris flow is more like a flood than a landslide and may knock down and wash away trees, houses, and even villages. In the above-quoted example, the features of debris flow are mapped onto the economic blow with the metaphor “ECONOMIC SHOCK/BLOW IS DEBRIS FLOW.”

Prior studies have shown natural disaster frames prevalent in English (de los Ríos, 2010), Spanish (Charteris-Black and Ennis, 2001), and German (Charteris-Black and Musolff, 2003) economic crises metaphors. The findings of the current study reveal that metaphors for economic decline in Mandarin have a similar tendency to use natural disaster frames. We could infer a concluding metaphor for economic crises or decline based on the five specific mapping analyses between natural disaster frames and economic decline presented above: ECONOMIC DECLINE/CRISES IS NATURAL DISASTERS.

First, it is reasonable to believe the metaphor that “economic decline/crises” refers to “natural disasters” because of the sudden, unpleasant, and sometimes catastrophic nature of natural disasters. This metaphor helps to remind us of the actual harm done by such events. These views confirm the following reality: Selection and salience are the two main components of framing (Entman, 1993). Five of the 10 frames for natural disasters mentioned above illustrate the metaphor that economic decline and crises is understood through natural disasters’ mapping. The destructive aspects of the five metaphorical frames in the economy domain are chosen and become prominent in their respective contexts, making the metaphors for economic decline accessible through frames of natural disasters.

Second, with all these five natural disaster frames employed to elaborate economic decline, different frames accentuate different aspects of the economy, which help compose a larger, if not all, picture of economic activities. For example, the earthquake frame emphasizes the shock on the economy; tsunami and flood emphasize the velocity of impact on the crises-stricken areas; landslides and debris flow imply the recessed and downward qualities of an economic crises. Semino (2021) has pointed out that metaphors can be deceptive and prevaricating, and they can also be enlightening and comforting. Therefore, the appropriateness of a metaphorical frame depends greatly on the communicator, context, purpose, and audience (Semino et al., 2018) and also depends on different frames to construct every complicated aspect of the target, especially in the case of constantly developing and multifaceted economic activities. It is exactly the diverse frames and diversity of language that display the complexity of the target. Moreover, the complexity of targets also calls for various metaphorical frames and linguistic expressions.

Third, as observed from Table 1, the following five frames could be adopted to describe economic decline, primarily economic crises: earthquake, flood, landslide, tsunami, and debris flow. The “LANDSLIDE” frame has the highest percentage among the five frames, with 2013 metaphorical frequencies (53.5%) and 1,372 (36.5%) economic crises metaphors in the collocation of “经济滑坡” (economic landslide). 海啸 (hǎi xiào) “TSUNAMI” frame comes in second when framing economic crises, with 60 (3.8%) frequencies in total 1,592 frequencies in CCL. The landslide frame is overwhelmingly higher than the tsunami frame in metaphorical economic uses. The reason for the prominent salience of the landslide frame in framing metaphorical negative impact among other natural disaster frames might lie in the fact that a landslide provides us with a vivid picture of sliding from a slope in sudden and downward movements of land in Mandarin, with severe damage caused to landslide-stricken areas. Metaphorically, a landslide’s rapid downward movements and its aftermath are perfectly mapped onto the decline and plummet of the economy, which might be the result of its salience in conveying economic crises, and that is in line with embodied cognition and conceptual metaphor theory (Lakoff and Johnson, 1980; Johnson, 1987; Lakoff, 1987).

As 滑坡 (huá pō) “LANDSLIDE” frame takes up the overwhelmingly biggest percentage of its total usage, we decided to involve it in the following experimental study: whether literal and landslide-framed economic crises bring different conceptualizations and lead to differences in opinions and decision-making.

## 3. An experimental study: opinions and decision-making in literal and landslide frames

### 3.1. Methodology

As the aforementioned discussion shows, among the above 10 discussed natural disaster frames, both overall metaphorical (53.5%) and economic metaphorical usages (36.5%) of landslide frame rank the highest, overwhelmingly and significantly higher than the second highest in economic crises metaphor—tsunami frame (3.8%). Based on the overwhelming percentage and difference between these two frames, we intended to further our metaphorical framing effect study by comparing landslide and literal frames in this section. We had conducted a pilot study and received desirable results before we carried out this experimental study.

#### 3.1.1. Participants

We had Chinese native speakers as our participants for the high percentage of landslide frames elicited from a Chinese corpus CCL. Given this metaphorical frame that prevails in our daily news and reports, we decided to recruit participants of various ages and walks of life, including students from majors closely related to economics and participants from various jobs such as doctors, teachers, office workers, and businessmen. A total of 416 Chinese with complete Mandarin literacy and comprehension (132 males and 284 females; age range from 18 to 58 years) recruited online from China participated in the experiment for monetary compensation, with 207 (*M*_age_ = 31.8, SD = 8.1) participants under the literal-framed condition and 209 (*M*_age_ = 28.5, SD = 7.4) under the landslide-framed condition. They were randomly assigned to either condition. Participation was voluntary, and the experimental protocol was approved by the independent ethics committee of Yangzhou University.

#### 3.1.2. Materials and procedure

This study adopted a self-paced reading paradigm. The participants were presented with an online survey on the platform Wenjuanxing (an equivalence to Qualtrics). Participants’ socio-demographic details, such as gender, age, or educational level, were collected. They were presented with a short Chinese paragraph of economic crises descriptions (either in the frame of literal or landslide; see Appendix). Each participant was randomly presented with literal or landslide-framed texts and required to finish a Likert scale with several questions to elicit opinions and decision-making. The two versions of the text were identical except in the literal and landslide frames in describing the shared topic: the economic crises. It was worth noting that the fabricated texts and questions prove to be credible, logical, and not self-contradictory, according to professionals in economics.

Every participant was asked three questions in Mandarin concerning their opinions and decision-making after reading the text: *(1)* Will you save more? *(2)* Will you worry about your financial situation? *(3)* Will factories face closure? *Provide a number from 1 to 5 (1 = “strongly disagree” and 5 = “strongly agree”).*

### 3.2. Results and discussion

In this section, we aimed to look at the possible effects of the use of literal and landslide frames in expressing economic crises and to see whether the use of these two frames would lead to different financial opinions, judgments, and behaviors.

**Figure 1:**
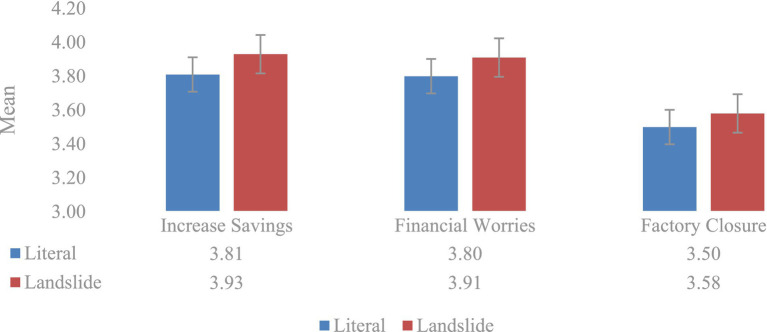
Mean of opinions and decisions in literal and landslide frames.

To the first question, (1) *Will you save more?*, we intended to investigate participants’ future behaviors when confronted with an economic crises in two different frames. The participants who read the text with the landslide frame seemed to respond more positively (*M* = 3.93, SD = 0.83), than the participants who read the text with the literal (*M* = 3.81, SD = 0.96), as shown in Figure 1. However, when comparing the scores with Student’s *t*-test to the independent samples, we observed that this difference was not statistically significant (*p* = 0.15).

Regarding the second question, (2) *Will you worry about your financial situations?*, we aimed to explore participants’ psychological states when confronted with an economic crises in two different frames so as to elicit corresponding judgments and behaviors. As shown in Figure 1, the participants who read the text with the landslide frame seemed to be more likely to worry about their financial situation (*M* = 3.92, SD = 0.89) than the participants who read the text with the literal frame (*M* = 3.80, SD = 0.96). However, this difference was not statistically significant (*p* = 0.22).

With regard to the third question, (3) W*ill factories face closure?*, we wanted to find out whether there was any difference in participants’ judgments toward financial trends when encountering two differently framed passages concerning the economic crises. As shown in Figure 1, the participants who read the text with the landslide frame seemed to be more likely to believe that more factories would face closure (*M* = 3.58, SD = 0.95) than the participants who read the text with the literal frame (*M* = 3.50, SD = 1.00). However, this difference was not statistically significant (*p* = 0.40).

In prior studies, it was confirmed that metaphorical frames shape reasoning and thought and affect people’s opinions and decisions by instantiating frame-consistent knowledge structures and inviting structurally consistent inferences (e.g., Charteris-Black, 2011; Thibodeau and Boroditsky, 2011, 2013, 2015; Boeynaems et al., 2017; Thibodeau et al., 2017; Hart, 2021; Benczes and Ságvári, 2022; Brugman et al., 2022; Tao et al., 2023). Landslide, as a natural disaster frame, is characterized by downward gestures at great velocity, severe mud and rock congestion in traffic, and bringing damage to areas nearby and underneath. Meanwhile, the landslide frame activates and embraces landslide-congruent qualities and landslide-consistent opinions, decisions, and even solutions.

For the severe and damaging qualities conferred by a landslide, a landslide-framed economic crises increases participants’ estimation of severity, manifested in their decisions and judgments regarding savings, financial worries, and factory closure estimation. Although the difference between literal and landslide-framed texts is not statistically significant, the metaphorical frame in this case indeed affected the way people perceived and felt about the economic crises, which is in line with the weak metaphorical framing effect.

## 4. General discussion

This study detailed concrete natural disaster frames frequently employed in economic crises metaphors in Mandarin. It is confirmed that five natural disaster frames (earthquake, flood, landslide, tsunami, and debris flow) played a great role in understanding economic decline to varying degrees, among which the landslide frame ranked the highest in Mandarin. Landslide frame stood out in the 10 frames for its highest overall metaphorical uses (53.5%) and metaphorically framed economic crises (36.5%) in the total frequency in CCL. These corpus-based findings contribute to the following three aspects. First, it provides a detailed and systematic combing of natural disaster frames of economic decline metaphors in Mandarin, which is in contrast to prior studies of frames such as *drought, whirlpool*, and *tornadoes* in English, German, Russian, etc. Evidence from both Mandarin and other languages draws a larger picture of humanity’s metaphorical language. Second, the natural disaster-framed economic crises is in line with conceptual metaphor theory and embodied philosophy (Lakoff and Johnson, 1980; Kövecses, 1986; Johnson, 1987; Lakoff, 1987). Humanity’s languages are teemed with metaphorical frames deriving from perceptible concepts and sensorimotor experiences of daily life, such as weather phenomena and natural disasters. Third, corpus-based findings provide a credible statistical analysis of the language database for the following metaphorical framing effect exploration of this study.

When confronted with landslide-framed and literal-framed economic crises texts, the vignette of the landslide-framed economic crises aroused more perceptions of its severity in economic crises. Therefore, participants demonstrated more willingness to save, more worries about their own financial situation, and a higher estimation of factories being closed. In spite of the statistically insignificant differences in opinions and decisions, the landslide-framed economic crises did confer a different way of perceiving and feeling about the economic crises when compared with the economic crises in the literal frame. The comparisons between two differently framed text responses demonstrated that participants tended to respond and behave in accordance with the metaphors they were exposed to. When the economic crises is framed in terms of a landslide, features of the metaphorical source domain (landslide) are mapped onto the target (economic crises). The target (economic crises) takes on features of the source (landslide): downward movement with great velocity and the great damage caused, which vividly depicts the aftermath of an economic crises.

Theoretically, the weak metaphorical framing effect exhibited in the landslide-framed economic crises is in line with prior studies that metaphorical frames would indeed influence reasoning, emotions, judgments, and behaviors to a certain degree (Thibodeau and Boroditsky, 2011, 2013, 2015; Boeynaems et al., 2017; Thibodeau et al., 2017; Hendricks et al., 2018; Hart, 2021; Benczes and Ságvári, 2022; Brugman et al., 2022; Tao et al., 2023). Practically, given that risk communication in different metaphorical frames influences the audience’s reasoning, decisions, and even behavior because of the framing effect (Gibbs and Cameron, 2008; Ervas et al., 2021), governments, reporters, and experts should be more cautious about metaphor choice in risk warning and communication. Semino (2021) has pointed out that metaphors can be deceptive and prevaricating, and they can also be enlightening and comforting. Therefore, the appropriateness of a metaphorical frame depends greatly on the communicator, context, purpose, and audience (Semino et al., 2018). In the moment of upcoming crises, effective, concise, clear, and official communication of risk sources, symptoms, and confident solutions is urgently needed (Hyland-Wood et al., 2021), and the metaphors used in risk reports should be in the service of effective and clear risk or crises communication.

There are certainly a couple of limitations to this study. The corpus-based analysis is limited to CCL, which might lead to the omission of some interesting metaphorical uses of natural disaster frames. Moreover, although the severity of the economic crises posed by landslide was greater than the literal frame and some weak metaphorical framing effects were detected and identified, the severity and framing effect were not significantly different. In light of these limitations, a venue for future research could be proposed. Further studies could consider investigating other factors functioning in metaphor processing, judgments, and decision-making, such as the role of age, gender, and socio-economic status. Meanwhile, the comparative study of metaphorical frames of economic crises in English and Mandarin is worth our further research. Moreover, to gain a clearer understanding of the metaphorical framing effect, some comparisons of economic crises metaphors in landslide and the oft-mentioned DISEASE frames could be further carried out to identify a greater and more comprehensive metaphorical framing effect. Meanwhile, the role metaphors play in risk communication deserves further and comprehensive study.

## 5. Conclusion

To conclude, our study confirmed a great number of natural disaster frames conveying an understanding of economic decline in Mandarin and offered a detailed analysis of the top 10 natural disaster frames in the corpus in question. Moreover, the landslide frame stands out in conceptualizing economic decline from all the 10 frames for its highest overall metaphorical uses (53.5%) and metaphorically framing economic crises (36.5%) in the total uses in CCL. The metaphorical framing effect exhibited in comparison between the Mandarin literal-framed and landslide-framed economic crises showcases that metaphorical frames impact the audience’s opinions, judgments, and even behaviors. The results inform government, journalists, and experts of the metaphor choices in risk warning and crises communication. We personally believe that metaphorical frame choice should depend on the degree of severity of the risk or crises and be honest with reality to avoid sending the general audience into panic and making rapid and extreme decisions. Furthermore, both communicators and the audience should be aware that metaphors are not mere figurative devices. Rather, metaphors, in various possible frames, might have the power to influence the way we think and behave in different ways.

## Data availability statement

The original contributions presented in the study are included in the article/supplementary material, further inquiries can be directed to the corresponding author.

## Author contributions

YZ: Writing – original draft. WY: Supervision, Writing – review & editing.

## Appendix

### Literally-framed economic crises description (English translation is not presented in the experiment)

过去5年来, 谷城地区的经济持续发展。但是近一年来, 谷城地区经济增长缓慢, 经济状况很不乐观, 地区生产总值同比下降了7%, 成为GDP出现负增长的地区之一。经济增长缓慢影响着各行各业, 导致生产和消费下降。而且销售业绩不佳, 商品过剩, 市场流动性较差, 人们生活水平下降, 某些行业影响较大, 发展减缓, 经济面临巨大挑战。.

(Translation: *Over the past 5 years, the economy of Gucheng Region has continued to grow. However, in the last year, the economy of Gucheng has been growing slowly and the economic situation is very bleak, with the regional GDP falling by 7% year-on-year, making it one of the regions with negative GDP growth. The slow economic growth has affected all sectors, leading to a decline in production and consumption. And with poor sales performance, a surplus of goods, less liquid markets and a much lower standard of living for people, certain sectors are more affected, growth is slowing and the economy is facing huge challenges*).

### Landslide-framed economic crises description (English translation is not presented in the experiment)

过去 5 年来, 谷城地区的经济持续发展。但是近一年来, 谷城地区正经历着经济大滑坡, 地区生产总值同比下降了7%, 成为GDP出现负增长的地区之一。经济像滑坡一样整体下滑, 冲刷着各行各业, 导致生产和消费下跌。而且商品销售停滞不前, 产品堆积, 市场的流动受到阻塞, 人们生活水平下滑, 某些行业受灾严重, 损失惨重, 经济或将跌至谷底。.

(*Translation: Over the past 5 years, the economy of Gucheng Region has continued to grow. However, in the last year, Gucheng is experiencing a major economic downturn, with GDP falling by 7% year-on-year, making it one of the regions with negative GDP growth. The economy as a whole has slumped like a landslide, violently washing over all sectors and causing production and consumption to keep falling. Moreover, sales of goods are stagnant, products are piling up, the flow of markets is blocked, people’s living standards are in decline, certain sectors are affected and losses are heavy, and the economy may hit rock bottom*).

## References

[ref1] Benczes R. Ságvári B. (2022). Migrants are not welcome: metaphorical framing of fled people in Hungarian online media, 2015–2018. J. Lang. Polit. 21, 413–434. doi: 10.1075/jlp.20042.ben

[ref2] Boeynaems A. Burgers C. Konijn E. A. Steen G. J. (2017). The effects of metaphorical framing on political persuasion: a systematic literature review. Metaphor. Symb. 32, 118–134. doi: 10.1080/10926488.2017.1297623

[ref3] Brugman B. C. Droog E. Reijnierse W. G. Leymann S. Frezza G. Renardel de Lavalette K. Y. (2022). Audience perceptions of COVID-19 metaphors: the role of source domain and country context. Metaphor. Symb. 37, 101–113. doi: 10.1080/10926488.2021.1948332

[ref4] Charteris-Black J. (2000). Metaphor and vocabulary teaching in ESP economics. Engl. Specif. Purp. 19, 149–165. doi: 10.1016/S0889-4906(98)00025-8

[ref5] Charteris-Black J. (2011). Politicians and rhetoric: the persuasive power of metaphor New York: Palgrave Macmillan.

[ref6] Charteris-Black J. (2021). Metaphors of coronavirus: Invisible enemy or zombie apocalypse?. Cham: Palgrave Macmillan

[ref7] Charteris-Black J. Ennis T. (2001). A comparative study of metaphor in Spanish and English financial reporting. Engl. Specif. Purp. 20, 249–266. doi: 10.1016/S0889-4906(00)00009-0

[ref8] Charteris-Black J. Musolff A. (2003). ‘Battered hero’ or ‘innocent victim’? A comparative study of metaphors for euro trading in British and German financial reporting. Engl. Specif. Purp. 22, 153–176. doi: 10.1016/S0889-4906(02)00012-1

[ref10] de los Ríos M. E. C. (2010). Cognitive devices to communicate the economic crisis: an analysis through covers in the economist. Ibérica 20, 1139–7241.

[ref9001] de Severino L. C. Israel D. A. Zonana V. G. (2001). Globalisation for beginners in Argentina: a cognitive approach. Amsterdam studies in the theory and history of linguistic science series. 4, 215–234.

[ref11] de Saint Preux A. D. Blanco O. M. (2021). The power of conceptual metaphors in the age of pandemic: the influence of the WAR and SPORT domains on emotions and thoughts. Lang. Commun. 81, 37–47. doi: 10.1016/j.langcom.2021.08.003

[ref9002] Elias S. Alderton D. (2020). Encyclopedia of geology. Academic Press.

[ref12] Entman R. (1993). Framing: toward clarification of a fractured paradigm. J. Commun. 43, 51–58. doi: 10.1111/j.1460-2466.1993.tb01304.x

[ref13] Ervas F. Rossi M. G. Ojha A. Indurkhya B. (2021). The double framing effect of emotive metaphors in argumentation. Front. Psychol. 12:628460. doi: 10.3389/fpsyg.2021.628460, PMID: 34194355 PMC8236609

[ref14] Flusberg S. J. Lauria M. Balko S. Thibodeau P. H. (2020). Effects of communication modality and speaker identity on metaphor framing. Metaphor. Symb. 35, 136–152. doi: 10.1080/10926488.2020.1767336

[ref15] Flusberg S. J. Matlock T. Thibodeau P. H. (2017). Metaphors for the war (or race) against climate change. Environ. Commun. 11, 769–783. doi: 10.1080/17524032.2017.1289111

[ref16] Gibbs R. W. Jr. Cameron L. (2008). The social-cognitive dynamics of metaphor performance. Cogn. Syst. Res. 9, 64–75. doi: 10.1016/j.cogsys.2007.06.008

[ref17] Pragglejaz Group (2007). MIP: a method for identifying metaphorically used words in discourse. Metaphor. Symb. 22, 1–39. doi: 10.1080/10926480709336752

[ref18] Hart C. (2021). Animals vs. armies: resistance to extreme metaphors in anti-immigration discourse. J. Lang. Polit. 20, 226–253. doi: 10.1075/jlp.20032.har

[ref19] Hendricks R. K. Demjén Z. Semino E. Boroditsky L. (2018). Emotional implications of metaphor: consequences of metaphor framing for mindset about cancer. Metaphor. Symb. 33, 267–279. doi: 10.1080/10926488.2018.1549835

[ref20] Hyland-Wood B. Gardner J. Leask J. Ecker U. K. (2021). Toward effective government communication strategies in the era of COVID-19. Human. Soc. Sci. Commun. 8, 1–11. doi: 10.1057/s41599-020-00701-w

[ref21] Johnson M. (1987). The body in the mind: the bodily basis of meaning, imagination, and reason. Chicago: University of Chicago Press

[ref22] Joris W. d’Haenens L. Gorp B. V. (2019). The effects of metaphorical frames on attitudes: the euro crisis as war or disease? Communications 44, 447–468. doi: 10.1515/commun-2018-2021

[ref23] Kövecses Z. (1986). Metaphors of anger, pride and love: a lexical approach to the structure of concepts. Amsterdam: John Benjamins.

[ref24] Lakoff G. (1987). Women, fire, and dangerous things 10. Chicago: University of Chicago press

[ref25] Lakoff G. Johnson M. (1980). Metaphors we live by Chicago: University of Chicago Press.

[ref26] Landau M. J. Keefer L. A. Rothschild Z. K. (2014). Epistemic motives moderate the effect of metaphoric framing on attitudes. J. Exp. Soc. Psychol. 53, 125–138. doi: 10.1016/j.jesp.2014.03.009

[ref27] Liu M. Chen J. (2023). Communicating telecom fraud risk in anti-telefraud messages: the effects of metaphorical frames on attitudes. Front. Psychol. 13:1093933. doi: 10.3389/fpsyg.2022.1093933, PMID: 36726509 PMC9885205

[ref28] López A. M. R. Llopis M. Á. O. (2010). Metaphorical pattern analysis in financial texts: framing the crisis in positive or negative metaphorical terms. J. Pragmat. 42, 3300–3313. doi: 10.1016/j.pragma.2010.06.001

[ref29] Morris M. W. Sheldon O. J. Ames D. R. Young M. J. (2007). Metaphors and the market: consequences and preconditions of agent and object metaphors in stock market commentary. Organ. Behav. Hum. Decis. Process. 102, 174–192. doi: 10.1016/j.obhdp.2006.03.001

[ref30] Pan Z. Kosicki G. M. (1993). Framing analysis: an approach to news discourse. Polit. Commun. 10, 55–75. doi: 10.1080/10584609.1993.9962963

[ref31] Piromalli E. (2021). Alienation, ideology, and power in the metaphors depicting the economic crisis in the media. Int. J. Commun. 15, 1932–8036.

[ref32] Reali F. Soriano T. Rodríguez D. (2016). How we think about depression: the role of linguistic framing. Revist. Latinoam. Psicol. 48, 127–136. doi: 10.1016/j.rlp.2015.09.004

[ref33] Ritchie L. D. (2013). Metaphor. Cambridge: Cambridge University Press.

[ref34] Robins S. Mayer R. E. (2000). The metaphor framing effect: metaphorical reasoning about text-based dilemmas. Discourse Process. 30, 57–86. doi: 10.1207/S15326950dp3001_03

[ref35] Schlesinger M. Lau R. R. (2000). The meaning and measure of policy metaphors. Am. Polit. Sci. Rev. 94, 611–626. doi: 10.2307/2585834

[ref36] Semino E. (2021). “Not soldiers but fire-fighters” – metaphors and COVID-19. Health Commun. 36, 50–58. doi: 10.1080/10410236.2020.1844989, PMID: 33167731

[ref37] Semino E. Demjén Z. Demmen J. (2018). An integrated approach to metaphor and framing in cognition, discourse, and practice, with an application to metaphors for cancer. Appl. Linguis. 39, 625–645. doi: 10.1093/applin/amw028

[ref38] Sopory P. Dillard J. P. (2002). The persuasive effects of metaphor: a meta-analysis. Hum. Commun. Res. 28, 382–419. doi: 10.1111/j.1468-2958.2002.tb00813.x

[ref9003] Steen G. J. Reijnierse W. G. Burgers C. (2014). When do natural language metaphors influence reasoning? A follow-up study to Thibodeau and Boroditsky. PloS one 9:e113536. 25490704 10.1371/journal.pone.0113536 PMC4260786

[ref39] Tang C. (2022). Amber Alert’or ‘heatwave warning’: the role of linguistic framing in mediating understandings of early warning messages about heatwaves and cold spells. Appl. Linguis. 43, 227–248. doi: 10.1093/applin/amab020

[ref40] Tao R. Kim S. J. Lu L. Kang J. McLeod D. (2023). Fighting fire or fighting war: examining the framing effects of COVID-19 metaphors. Health Commun. 38, 1–15. doi: 10.1080/10410236.2023.2253398, PMID: 37661328

[ref41] Thibodeau P. (2017). The function of metaphor framing, deliberate or otherwise, in a social world. Metaphor Social World 7, 270–290. doi: 10.1075/msw.7.2.06thi

[ref42] Thibodeau P. H. Boroditsky L. (2011). Metaphors we think with: the role of metaphor in reasoning. PLoS One 6:e16782. doi: 10.1371/journal.pone.0016782, PMID: 21373643 PMC3044156

[ref43] Thibodeau P. H. Boroditsky L. (2013). Natural language metaphors covertly influence reasoning. PLoS One 8:e52961. doi: 10.1371/journal.pone.0052961, PMID: 23301009 PMC3534638

[ref44] Thibodeau P. H. Boroditsky L. (2015). Measuring effects of metaphor in a dynamic opinion landscape. PLoS One 10:e0133939. doi: 10.1371/journal.pone.0133939, PMID: 26218229 PMC4517745

[ref45] Thibodeau P. H. Flusberg S. J. (2017). Metaphorical accounting: how framing the federal budget like a household’s affects voting intentions. Cogn. Sci. 41, 1168–1182. doi: 10.1111/cogs.12475 28139843

[ref46] Thibodeau P. Hendricks R. K. Boroditsky L. (2017). How linguistic metaphor scaffolds reasoning. Trends Cogn. Sci. 21, 852–863. doi: 10.1016/j.tics.2017.07.001, PMID: 28789831

[ref47] Wang J. A. (2006). Spatio-temporal pattern of natural disasters in China. Beijing: Science Press.

[ref48] Wang H. Runtsova T. Chen H. (2013). Economy is an organism–a comparative study of metaphor in English and Russian economic discourse. Text Talk 33, 259–288. doi: 10.1515/text-2013-0012

[ref49] World Health Organization (2020). Risk communication and community engagement readiness and response to coronavirus disease (COVID-19): Interim guidance, 19 March 2020 (No. WHO/2019-nCoV/RCCE/2020.2). Geneva: World Health Organization.

[ref50] Zeng W. H. Burgers C. Ahrens K. (2021). Framing metaphor use over time: ‘free economy’ metaphors in Hong Kong political discourse (1997–2017). Lingua 252:102955. doi: 10.1016/j.lingua.2020.102955

[ref51] Zhang C. Lin Z. Jin S. (2022). What Else besides war: deliberate metaphors framing COVID-19 in Chinese online newspaper editorials. Metaphor. Symb. 37, 114–126. doi: 10.1080/10926488.2021.1948333

